# Dinoflagellate vertical migration fuels an intense red tide

**DOI:** 10.1073/pnas.2304590120

**Published:** 2023-08-28

**Authors:** Bofu Zheng, Andrew J. Lucas, Peter J. S. Franks, Tamara L. Schlosser, Clarissa R. Anderson, Uwe Send, Kristen Davis, Andrew D. Barton, Heidi M. Sosik

**Affiliations:** ^a^Scripps Institution of Oceanography, University of California San Diego, La Jolla, CA 92093; ^b^Department of Mechanical and Aerospace Engineering, University of California San Diego, La Jolla, CA 92093; ^c^Southern California Coastal Ocean Observing System, Scripps Institution of Oceanography, University of California San Diego, La Jolla, CA 92093; ^d^Department of Earth System Science, University of California, Irvine, CA 92697; ^e^Department of Civil and Environmental Engineering, University of California, Irvine, CA 92697; ^f^Department of Ecology, Behavior and Evolution, University of California San Diego, La Jolla, CA 92093; ^g^Biology Department, Woods Hole Oceanographic Institution, Woods Hole, MA 02543

**Keywords:** harmful algal blooms, Oceanography, coastal water quality

## Abstract

Harmful algal blooms (HABs) are increasing globally, causing economic, human health, and ecosystem harm. In spite of the frequent occurrence of HABs, the mechanisms responsible for their exceptionally high biomass remain imperfectly understood. A 50-y-old hypothesis posits that some dense blooms derive from dinoflagellate motility: organisms swim upward during the day to photosynthesize and downward at night to access deep nutrients. This allows dinoflagellates to outgrow their nonmotile competitors. We tested this hypothesis with in situ data from an autonomous, ocean-wave-powered vertical profiling system. We showed that the dinoflagellate *Lingulodinium polyedra*’s vertical migration led to depletion of deep nitrate during a 2020 red tide HAB event. Downward migration began at dusk, with the maximum migration depth determined by local nitrate concentrations. Losses of nitrate at depth were balanced by proportional increases in phytoplankton chlorophyll concentrations and suspended particle load, conclusively linking vertical migration to the access and assimilation of deep nitrate in the ocean environment. Vertical migration during the red tide created anomalous biogeochemical conditions compared to 70 y of climatological data, demonstrating the capacity of these events to temporarily reshape the coastal ocean’s ecosystem and biogeochemistry. Advances in the understanding of the physiological, behavioral, and metabolic dynamics of HAB-forming organisms from cutting-edge observational techniques will improve our ability to forecast HABs and mitigate their consequences in the future.

## Significance

Extremely dense harmful algal blooms (HABs) are an increasing problem globally. How microscopic, single-celled organisms can reach such high abundances is still poorly understood. Over 50 y ago, it was postulated that motile dinoflagellates could form dense blooms through their vertical swimming, which gave them a competitive advantage over other phytoplankton. We tested this hypothesis with innovative in situ observations during a dense bloom. The motile dinoflagellates swam downward at night into the deep nutrient pool, and upward during the day to photosynthesize. Decreases of nutrients in deep waters were proportional to increases in organism abundance, directly linking organism behavioral and metabolic activities. These observations demonstrate that vertical migration is central to the formation of exceptionally dense dinoflagellate HABs.

Harmful algal blooms (HABs) are a global problem, impacting human health, ecosystem function, and water quality. HAB outbreaks are estimated to cost at least tens of millions of dollars annually in the United States alone (1, 2). Major strides have been made in elucidating the biochemical pathways for toxin production, developing monitoring, tracking, and response strategies in key locations, and improving modeling capacity (3–5). However, the biotic and abiotic factors that lead to the extreme biomass density of HABs are not yet fully understood, complicating efforts to forecast their occurrence and impact (6–8).

Dinoflagellate blooms often occur during strongly stratified ocean conditions (9). Nutrient concentrations in the upper, relatively warm, sunlit waters become depleted through uptake by the phytoplankton community. These upper waters are separated from the deeper, cold, dark, and nutrient-rich waters by a strong density gradient that inhibits the upward physical transport of nutrients. Many HAB-forming dinoflagellate species—like *Lingulodinium polyedra* (formerly *Lingulodinium polyedrum; Gonyaulax polyedra*)—are active swimmers (10–12). Early field and laboratory observations documented diel vertical migrations (DVMs) of dinoflagellates, in which organisms accumulated near the surface during daylight hours and swam to depth at night (13–15). Based on observational evidence provided by Holmes et al. (16), Eppley et al. (17) were the first to hypothesize that this migration behavior might allow dinoflagellates to form extremely dense blooms by gaining access to light at the surface during the day, and nutrients at depth during the night—deriving a competitive advantage over nonmotile photoautotrophic organisms during periods of strong, vertical stratification.

In the 50 y since Eppley et al. (17) was published, laboratory experiments have supported the DVM-bloom hypothesis, demonstrating that dinoflagellates can vertically migrate and that their migrations depend on light, nutrient distributions, stratification, and turbulence (e.g., refs. 17–20). However, despite extensive laboratory research, the DVM-bloom hypothesis has never been conclusively tested in the field.

Field confirmation of the DVM-bloom hypothesis requires evidence that the dinoflagellates vertically migrate, that the dinoflagellates take up nutrients from below the euphotic zone during this migration, and that dinoflagellate abundances increase in proportion to the nutrient uptake. The lack of conclusive field evidence of the DVM-bloom hypothesis is primarily due to two impediments: 1) the need for concurrent in situ measurements of biological, physical, nutrient, and light conditions, and 2) the requirement for those data to be collected with adequate resolution in time and space to separate variability due to physical forcing [e.g., currents, internal waves (IWs)] from variability mediated by biological processes. Here, we use data acquired by a moored autonomous profiler to provide field evidence testing—and supporting—the DVM-bloom hypothesis (17).

**Fig. 1.**
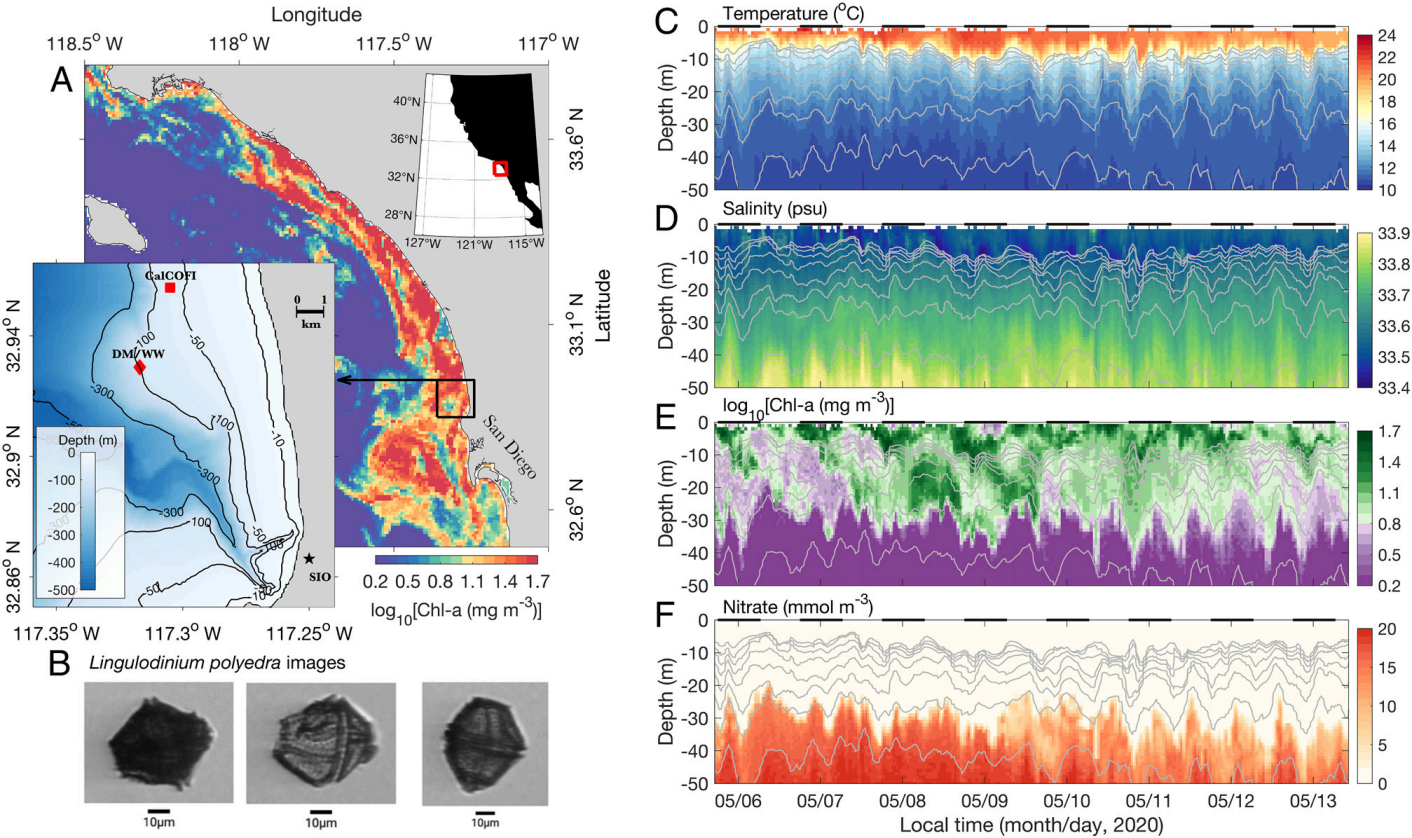
The 2020 *L. polyedra* red tide. (*A*) May 6, 2020 Visible Infrared Imaging Radiometer Suite (VIIRS)—Suomi National Polar-Orbiting Partnership 1-d composite satellite chlorophyll-a (Chl-a) image from the SCB (corresponding to the red box in the *Upper Right Inset* panel). The *Lower Left Inset* shows the bathymetric map of the study area (corresponding to the black box in the main panel), with the DM and the WW (red diamond), and the CalCOFI station (Line: 93.3, Station: 26.7) (red square). (*B*) Images of *L. polyedra* captured by the IFCB on the DM on May 7, 2020. (*C*–*F*) WW-measured time/depth series of temperature, salinity, Chl-a concentration, and nitrate concentration respectively, with isopycnals contoured as gray lines, ranging from 1,024.35 kg m^−3^ to 1,025.75 kg m^−3^, with an increment of 0.2 kg m^−3^. Black bars on the upper boundary of each panel indicate nighttime. Note that only 50-m depth and 7 d of data from the full record are shown here.

Blooms of the dinoflagellate *L. polyedra* have been fairly common in the Southern California Bight (SCB) for at least 120 y, often growing to cell densities high enough to change the color of the water during the day (hence the term “red tide,” ref. 21) and generate bioluminescence at night (22–26). An unusually intense *L. polyedra* red tide occurred in the SCB and Northern Baja California waters in the spring of 2020 (Fig. 1*A*). At its peak, the bloom reached nine million cells per liter at the Scripps Institution of Oceanography (SIO) Pier, with chlorophyll-a (Chl-a) concentrations of 897 mg m^−3^ on April 27 (27, 28). These are the highest values of Chl-a ever measured at Scripps Pier in nearly 40 y of monitoring, and represent roughly 1,000 times the average Chl-a value found in the region (27).

Near-surface images collected by an in situ Imaging FlowCytobot (IFCB, see *Methods*) at the Del Mar (DM) mooring (Fig. 1*B*) showed that *L. polyedra* biomass increased rapidly in early April, while other taxa did not (*SI Appendix*, Fig. S1). By early May, satellite-detected Chl-a concentrations were >50 mg m^−3^ (>50 times prebloom values; Fig. 1*A*). At this time, *L. polyedra* was the most abundant organism in the IFCB measurements, contributing >80% of the total plankton carbon biomass during daytime (*SI Appendix*, Fig. S1). At Scripps Pier, *L. polyedra* cell counts regularly exceeded 1 million cells l^−1^ in early May, with near-surface Chl-a concentrations over 90 mg m^−3^ (27). The decay of the bloom in mid-May (*SI Appendix*, Fig. S1) led to local anoxia and extensive fish and crustacean mortality in the region (29).

## Depth-Time-Resolved Measurements during the Red Tide

To investigate the interplay of biogeochemical patterns, organismal behavior, and physical dynamics during the peak of the *L. polyedra* bloom, we moored a Wirewalker (WW) ocean-wave-powered autonomous profiler adjacent to the DM mooring, ~4 km offshore coastal San Diego (Fig. 1*A*, *Methods*, refs. 30 and 31). The WW payload consisted of an integrated suite of sensors to simultaneously measure in situ nitrate, multispectral irradiance, biooptical water properties and hydrographic properties. The WW mooring yielded full-depth (100 m) profiles of light, nitrate concentration, Chl-a fluorescence, optical backscatter, temperature, salinity, and density at 1-m vertical resolution every 15 min for a period of 2 wk (*Methods*).

These data allowed us to quantify the relationships between dinoflagellate vertical migration, light, and nitrate concentration. Our analyses revealed the cyclic downward vertical migration of dinoflagellates at night, coincident with the loss of nitrate at depth, and proportional increases in the vertically integrated Chl-a and turbidity of the bloom. Toward the end of our data record, the deep nitrate pool was so depleted that the dinoflagellate bloom began to dissipate.

The 2020 bloom event began during unusual conditions in Southern California coastal waters. Anomalously high rainfall in March and April was followed by a prolonged sunny and calm period through mid-May—an uncommon springtime combination (*SI Appendix*, Fig. S2). The resulting cooccurrence of intense freshwater input and record heating led to record-high sea surface temperatures (>26 °C, https://cdip.ucsd.edu/), and strong subsurface stratification (Fig. 1 *C* and *D*)—conditions that are known to favor dinoflagellate bloom initiation (9, 32).

## Vertical Migration of Dinoflagellates

**Fig. 2.**
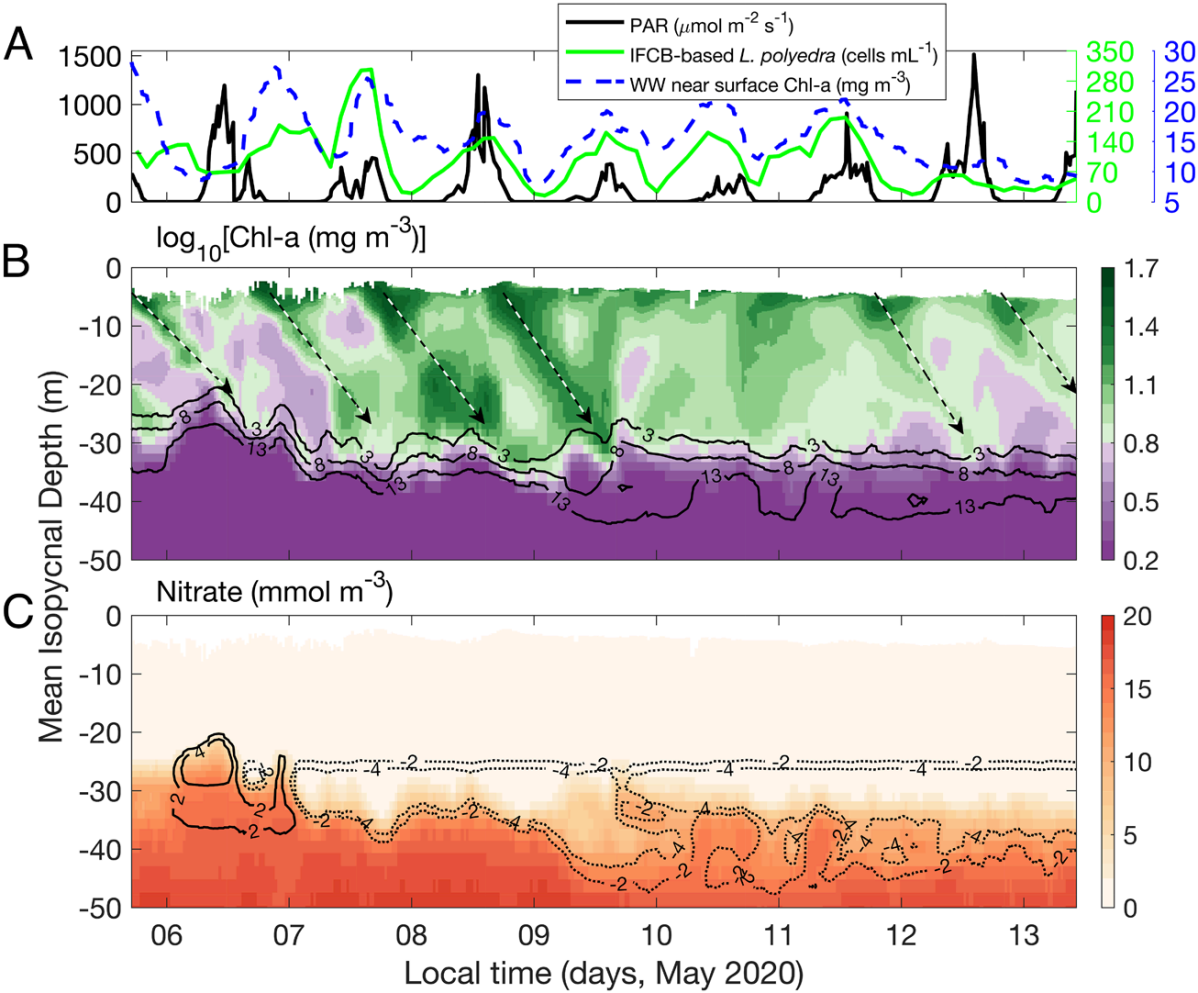
Time series of biogeochemical observations. (*A*) left *Y* axis: near-surface PAR estimated from the subsurface irradiance measurements on the WW profiler at 2-m depth. Right *Y* axis: IFCB-based *L. polyedra* cell concentrations and WW-measured near-surface chlorophyll concentrations, respectively. (*B*) and (*C*) along-isopycnal Chl-a, and nitrate concentrations from the WW. The black contours in B are the 3, 8, and 13 mmol m^−3^ isopleths of nitrate. The black-white dashed arrows indicate the dinoflagellate migration paths. The contours in C are nitrate anomalies referenced to the first profile during this period, with positive anomalies in black solid lines and negative anomalies in black dotted lines. Note that the *X* axis here is the local time, and the *Y* axis is the mean depth for each isopycnal (*SI Appendix*, section 6).

In the WW time series, periodically elevated Chl-a concentrations were observed down to ca. 30 to 40-m depth, tens of meters deeper than the measured euphotic zone (Fig. 2 and *SI Appendix*, Fig. S4), with patches of high Chl-a moving vertically across isopycnal surfaces with a near-daily rhythm (Fig. 1*E*). A daily rhythm was also apparent in the near-surface (~4 m depth) IFCB *L. polyedra* cell counts (high during the day, low at night), directly linking the WW Chl-a measurements with *L. polyedra* abundances (Fig. 2*A*). Over a week, nitrate became depleted at depths where Chl-a concentrations were periodically high, leading to the nitracline deepening by more than 10 m (Fig. 1*F*)—an abnormal situation for these coastal waters (see below; ref. 33).

Vertical motions from IWs—waves analogous to surface waves, but propagating along density surfaces in the ocean interior—caused ~20-m vertical fluctuations (peak-to-trough amplitude) of the Chl-a, nitrate, and density distributions (Fig. 1 *C–F*). Plotting the data in an isopycnal vertical coordinate removes these vertical motions (Fig. 2 and *SI Appendix*, section 6). For the remainder of this paper, all data are mapped onto isopycnals and plotted against the mean depths of the isopycnals as the vertical coordinate (*SI Appendix*, section 6). This transformation makes it clear that coherent patches of high Chl-a moved downward from the surface, across isopycnals, to >30-m depth with a ~daily periodicity (Fig. 2*B*). This descent of Chl-a was most obvious on sunny days (Fig. 2 *A* and *B*). Given the dominance of Chl-a by *L. polyedra* during the bloom (*SI Appendix*, Fig. S1), and the clear correlations of fluctuations in IFCB-based *L. polyedra* cell concentration and WW-measured Chl-a (Fig. 2*A*), these Chl-a patches most likely reflected layers of *L. polyedra* that were migrating vertically.

The *L. polyedra* organisms started their descent from the surface around sunset (6:00 PM local time; Fig. 2 *A* and *B*), moving downward at ~380 μm s^−1^—about 1.4 m h^−1^—often slowing at depth during the subsequent daylight hours. The dense dinoflagellate aggregations arrived at the maximum migration depth (30 to 40 m) after ~18 to 24 h of downward swimming (Fig. 2*B*). These swimming speeds closely match laboratory measurements of *L. polyedra* swimming (100 to 400 μm s^−1^; refs. 17, 34, and 35), and are 10 times greater than their sinking speeds (~35 μm s^−1^; ref. 16). On cloudy days with less incoming solar radiation (May 9 and May 10, 2020, Fig. 2*A*), the subsurface vertical migration was much less apparent (no coherent Chl-a paths), suggesting that the intensity of daily solar radiation is an important trigger for vertical migration.

## Subeuphotic Zone Nitrate Loss

The downward migration of the dinoflagellates terminated at the depth of the nitracline (strongest vertical gradient in nitrate concentration), where nitrate increased from undetectable levels to >10 mmol m^−3^ over only 2 to 3 m (Fig. 2*B*). At the beginning of our measurements, the nitracline was at a mean-isopycnal depth of ~30 m (Fig. 2*C*). Subeuphotic zone nitrate concentrations then decreased over time, resulting in a deepening of the nitracline from an isopycnal depth of ~30 m to ~40 m over a few days. Eventually, nitrate concentrations became undetectable at the maximum *L. polyedra* migration depth, far below the euphotic zone (Fig. 2*C* contours; *Methods*).

**Fig. 3.**
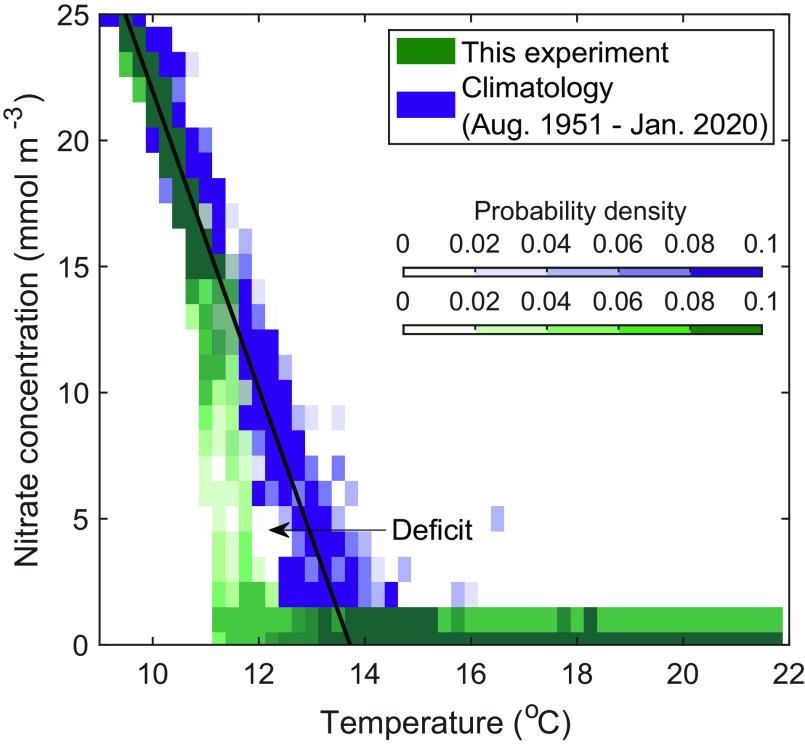
Nitrate-temperature relationship. The combined probability density function (PDF) of nitrate for each 0.25 °C temperature bin, with WW measurements from this study in green, and climatological data (70 y of CalCOFI bottle samples, line 93.3, station 26.7) in purple. The black solid line represents the least-squares fit to the climatological PDF for temperatures <14 °C, with the regression equation: Nitrate = −5.9 T+80.94 (T < 14 °C).

The loss of subeuphotic zone nitrate is apparent in comparisons of historical nitrate and temperature data to data gathered by the WW during the 2020 bloom. Measurements collected over 70 y (CalCOFI program, 1951 to 2020, *Methods*), 5 km from the study site, show the nitrate-temperature relationship to be extremely stable over time (Fig. 3), with a climatological relationship of the nitracline with the 14 °C isotherm (also in ref. 36). Our WW observations revealed a clear deficit of nitrate relative to those climatological values between temperatures of 11 °C and 14 °C during the bloom (Fig. 3). In the deeper, cold waters (<11 °C), the nitrate concentrations during the *L. polyedra* bloom were consistent with climatological values, suggesting that conditions remained typical for the area in the waters below the maximum migration depth of the dinoflagellates.

The nitrate deficit layer was roughly 25 m in vertical extent, extending from the base of the euphotic zone at ~15 m depth down to ~40 m depth. The maximum nitrate deficit for a given temperature was 14 mmol N m^−3^ relative to climatology (Fig. 3), reflecting a profound change in the distributions of inorganic nutrients during the bloom.

## Relating the Nitrate Deficit to Vertical Migration and Phytoplankton Growth

We hypothesized that the subeuphotic zone nitrate deficit was created through nitrate uptake by the downward-migrating dinoflagellates. To test this, we quantified the amount and rate of nitrate loss due to biological uptake, independent of fluctuations driven by physical forcing, such as horizontal and vertical advection of nitrate gradients due to coastal currents and IWs.

Transformation to an isopycnal coordinate reference frame removes vertical fluctuations due to IWs, as described above. To remove nitrate fluctuations driven by currents acting on existing horizontal nitrate gradients, we tracked nitrate changes in individual water parcels defined by their unique temperature and salinity properties (*SI Appendix*, section 8). The oscillating horizontal currents caused individual water parcels to appear multiple times on an isopycnal at the mooring, allowing us to track nitrate changes separately for each parcel as a function of depth and time. This technique largely removed the effects of horizontal advection of nitrate variability at the mooring location (see Fig. 2*C* contours), allowing us to obtain estimates of the depth- and time-dependent biologically driven loss rates of nitrate. It also allowed us to obtain error estimates for our calculations (*SI Appendix*, Eqs. **S9, S10, S12, S14,** and **S15**).

**Fig. 4.**
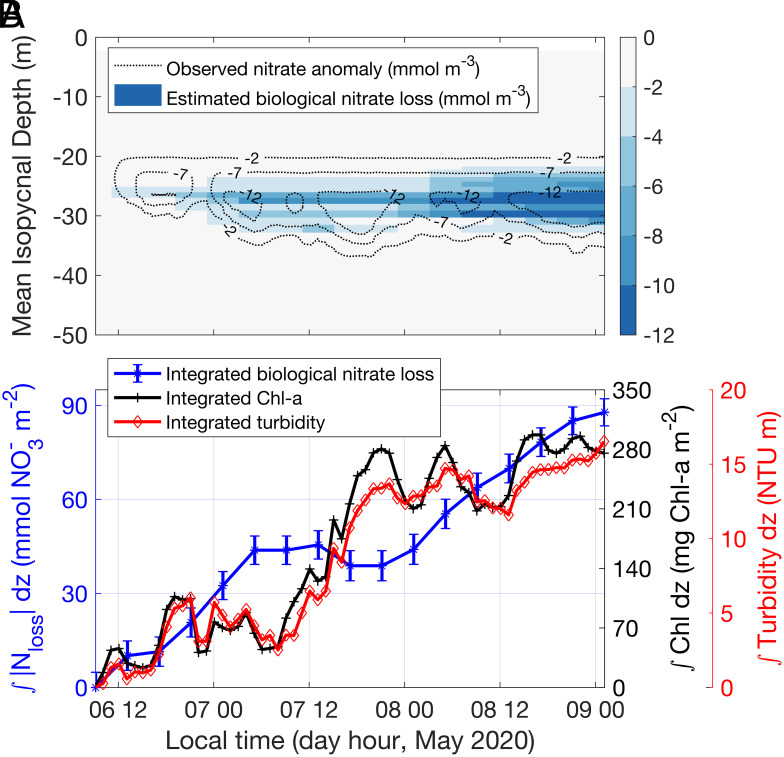
Variability in nitrate concentration at depth due to dinoflagellate migrations. (*A*) Estimated biologically induced nitrate loss (color) and observed nitrate anomaly (contours) referenced to the first profile during this period. (*B*) Time series of depth-integrated (0 to 33 m) absolute biologically induced nitrate loss (blue line with star markers), Chl-a concentrations (black line with plus markers), and turbidity (red line with diamond markers). Error bars represent error estimations following the error propagation procedures (*SI Appendix*).

Successive nitrate measurements in the same water masses allowed estimation of the biological nitrate loss rates, $\rho_{NO3}$ (mmol N m^−3^ d^−1^; *SI Appendix*, section 9). During a representative 2.5 d, from local noon on May 6th to local midnight on May 8th, the mode of nitrate loss rate was ~6 mmol N m^−3^ d^−1^ in the nitracline. The depth-integrated average nitrate loss rate was 31 ± 4 mmol N m^−2^ d^−1^ (434 ± 56 mg N m^−2^ d^−1^), which is 10 times greater than the previously estimated physical nitrogen supply rates over the inner shelf of SCB (36), and 20 times the vertical nitrate flux rate based on the Biologically Effective Upwelling Transport Index (37) at the sample time—emphasizing that the high nitrate demand during the red tide could not be met by typical physical nutrient delivery mechanisms. Integrating $\rho_{NO3}$ in time generates estimates of the amount of nitrate taken up by the dinoflagellates, $N_{loss}$ (mmol N m^−3^) (*SI Appendix*, section 9 and Fig. S9). $N_{loss}$ ranged from 0 to more than 12 mmol N m^−3^ in the ~10 to 13 m thick layer below the euphotic zone (Fig. 4*A*).

Fluctuations of nitrate in the layer of nitrate loss (Fig. 4*A*) coincide in time and space with the maximum migration depths of *L*. *polyedra* (Fig. 2*B*). Furthermore, the calculated nitrate loss rates are consistent with nitrate uptake rates reported from lab experiments with *L. polyedra* (1 to 4 mmol N m^−3^ d^−1^; ref. 38 and 39), and the estimated average N:Chl-a ratio of 5.3 is within the expected range for *L*. *polyedra* (40). Importantly, the time series of depth-integrated nitrate loss, $\int N_{loss}\left(z,t\right)dz$ , was significantly correlated with the time series of increases in both depth-integrated Chl-a concentration (R = 0.81, *P* < 0.0001) and depth-integrated turbidity measured at 532 nm (R = 0.82, *P* < 0.0001) (Fig. 4*B*)—directly linking the loss of nitrate to the gains of Chl-a and organic particle loads. The consistency of estimated nitrate loss rates with lab measurements of *L. polyedra*’s nitrate uptake rates, and the correlations of nitrate loss with increases in both Chl-a and turbidity, provide strong support for the hypothesis that the nitrate loss was caused by the uptake by *L. polyedra* as they migrated vertically into the subeuphotic zone nitrate pool. This nitrate uptake depleted the subeuphotic zone nitrate concentrations and deepened the nitracline (Fig. 4*A*). The measured descent rates of the Chl-a patches showed a maximum of ~35 m d^−1^ vertical travel; we suggest that the deepening of the nitracline eventually put it beyond the reach of the dinoflagellates’ daily migration, contributing to the eventual demise of the bloom.

## Summary

Eppley et al. (17) hypothesized that exceptionally dense blooms can form by dinoflagellates migrating upward during the day to photosynthesize, and downward at night to take up nitrate and other nutrients from the deep nutrient pool below the euphotic zone. Despite laboratory experiments supporting this DVM-bloom hypothesis, a comprehensive test of this hypothesis in the field has been lacking. With a moored, multisensor autonomous profiling vehicle deployed during an intense red tide in Southern California, we showed 1) that the bloom-forming dinoflagellates vertically migrated, 2) that they took up nutrients from below the euphotic zone during the deep phase of the migration, and 3) that dinoflagellate abundances subsequently increased in proportion to the nutrient uptake. These observations confirm that vertical migration played a key role in maintaining the extreme biomass of the bloom.

Vertical migration allows dinoflagellates to acquire essential nutrients from below the waters in the euphotic zone. This enables motile dinoflagellates to overcome the inverse depth gradients of light and nutrients, and provides them a competitive advantage over nonmotile species in strongly stratified conditions. In the case reported here, the nitracline was ultimately forced to a depth far below the euphotic zone, suggesting that nonmotile phytoplankton such as the typically dominant diatom species would be starved of inorganic nutrients at depths with sufficient sunlight for photosynthesis and growth. The dense, prolonged bloom we observed in 2020 produced historically anomalous subsurface nutrient deficits, showing the potential of HABs to reshape the environmental and biogeochemical character of the coastal ocean. The continued development of autonomous systems equipped with high-resolution physical and biogeochemical sensors will advance our understanding of the interplay between environmental conditions and the behavior and metabolic needs of bloom-forming organisms. Including these biophysical feedbacks in numerical models is necessary to improve our ability to forecast HABs.

## Methods

Our data were acquired by a moored ocean-wave-powered WW profiler, the long-term Del Mar Mooring (DM), the California Cooperative Oceanic Fisheries Investigations (CalCOFI) program, and the Southern California Coastal Ocean Observing System. The WW is an autonomous vertical profiling system that allows the collection of high-resolution upper-ocean physical and biogeochemical properties (30, 31, 41). The WW was equipped with a conductivity-temperature-depth sensor (RBR Concerto), nitrate sensor (Submersible Ultraviolet Nitrate Analyzer (SUNA V2), Sea-Bird Scientific (SBE)), Chl-a fluorescence and optical backscatter sensor (SBE ECOPuck), and irradiance sensor (SBE OCR-504). The SUNA estimates nitrate concentration (unit: μM, 1 μM = 1 mmol m^−3^) based on the absorption spectrum of the water sample in the ultraviolet (UV) light region (42). Data analysis procedures for raw nitrate data quality control, nonphotochemical quenching correction for fluorescence, calculation of photosynthetically available radiation (PAR), and estimation of turbidity are presented in Supplementary Information (*SI Appendix*, sections 3–5). The SUNA was configured to sample with a duty cycle of 20 min h^−1^ with a 1 Hz sampling rate. All other sensors onboard the WW had a sampling frequency of 6 Hz. Power demands of the sensor suite required battery replacement weekly. Biooptical sensors malfunctioned during the first week deployment and therefore are not analyzed here. Over the two week period from May 5, 2020 to May 21, 2020, the gridded WW data had 1-m vertical resolution and ~15-min temporal resolution, determined by the sampling rate of SUNA and vertical speed of the WW, respectively.

The DM mooring (Fig. 1*A*) is a full water column physical–biogeochemical mooring maintained by the Ocean Time-Series Group at the Scripps Institution of Oceanography. It continuously measures ocean temperature, salinity, currents, oxygen concentration, fluorescence, and nutrients. Phytoplankton images (Fig. 1*B*) were captured at approximately 4-m depth by an IFCB (43, 44)—an automated underwater imaging system that captures high-resolution images of suspended particles, including *L. polyedra*. IFCB data from the DM are available at https://ifcb-data.whoi.edu/timeline?dataset=SIO_Delmar_mooring. Images were automatically analyzed following approaches previously developed for IFCB data (44, 45, with update to version 4 products). Biovolume was determined for each plankton target in IFCB images according to the distance map algorithm of Moberg and Sosik (46). Each biovolume was converted to cell carbon following the relationships described by Menden–Deuer and Lessard (47), and then carbon concentration in each sample was determined by summing up contributions from all plankton targets with equivalent spherical diameter ≥5 μm and dividing by the volume of seawater imaged. The IFCB is sensitive enough to reliably quantify cells above the 5-μm lower limit and is able to image cells up to ~180-μm. Phytoplankton in each IFCB sample were identified to species or genus levels with automated image classification approaches (convolutional neural network with Inception v3 architecture, initialized with weights from ImageNet, and fine-tuned with human labeled IFCB images). Kahru et al. (27) examined the daily average abundance of *L. polyedra* inferred from the IFCB and found correspondence with nearby microscope counts and satellite-measured Chl-a during this red tide.

The CalCOFI program is a long-term ocean monitoring program that has collected full water-column samples at specific stations off the coast of California since 1949 (quarterly since 1984). We used data collected at the innermost station on Line 93.3, located at 32.956 ^o^N 117.305 ^o^W, ~5 km from the DM location.

The CalCOFI and WW nitrate vs. temperature PDFs (Fig. 3) were calculated with nitrate data in 0.25 °C temperature bins and 1 mmol m^−3^ nitrate bins.

To convert from depth to isopycnal coordinates, raw physical and biogeochemical variables from each individual WW profile were interpolated onto uniformly gridded density bins (density interval of 0.02 kg m^−3^) (*SI Appendix*, Figs. S5 and S6). Mean isopycnal depths were calculated as the time-averaged interpolated depth for each gridded density bin over the course of the study period (*SI Appendix*, section 6).

To remove the effects of horizontal along-isopycnal advection from biological processes, for each isopycnal, data were binned by their level of spice (Temperature/Salinity properties; ref. 48) with a 0.005-kg m^−3^ interval. Points in the same spice bin on an isopycnal were assumed to be the same water mass (*SI Appendix*, Fig. S8). The rates of change of nitrate ( $\rho_{NO3}$ , units: mmol N m^−3^ d^−1^) were estimated from nitrate concentration changes within each individual water mass by first fitting an exponential function to pairs of nitrate measurements separated by a time interval ∆t (determined by the reappearance of a particular water mass at the mooring): $N\left(t+∆t\right)=N\left(t\right)e^{r∆t}$ . These fits gave estimates of r—the nitrate-specific rate of change (units: d^−1^). The predicted biologically driven nitrate loss was then calculated by temporally iterating over a uniform time interval *δt* (details in *SI Appendix*, Fig. S9). The nitrate loss rate $\rho_{NO3}$ was determined by a temporal differentiation of the predicted nitrate loss (*SI Appendix*, Fig. S9). The associated error for each estimated quantity followed SE propagation procedures (*SI Appendix*).

## Supplementary Material

Appendix 01 (PDF)

## Data Availability

Wirewalker observational data for this paper are available through Dryad: https://doi.org/10.6076/D1SW2S (49). Del Mar mooring and CalCOFI data are available at https://mooring.ucsd.edu/delmar1/delmar1_15/ (50) and https://calcofi.org/data/oceanographic-data/bottle-database/ (51), respectively.
