# Supplementary material for: Dinoflagellate vertical migration fuels an intense red tide
Source: Proc Natl Acad Sci U S A. 2023 Aug 28;120(36):e2304590120. doi: 10.1073/pnas.2304590120 (PMC10483641; doi:10.1073/pnas.2304590120)
Supplement: Supplementary file 1 — Appendix 01 (PDF) [file pnas.2304590120.sapp.pdf]

Supporting Information for
Dinoflagellate vertical migration fuels an intense red tide

Bofu Zheng, Andrew J. Lucas*, Peter J.S. Franks, Tamara L. Schlosser, Clarissa Anderson, Uwe Send, Kristen Davis, Andrew D. Barton, Heidi M. Sosik

*Email: ajlucas@ucsd.edu

This PDF file includes:

Supporting text
Figures S1 to S10
SI References

Supporting Information Text

1. Biomass contribution from *Lingulodinium polyedra*

Biovolume and cell carbon were determined for each plankton target in IFCB images (Methods in the main text). Carbon concentration in each sample was determined by summing up contributions from all plankton targets with equivalent spherical diameter ≥ 5 μm and dividing by the volume of seawater imaged. Results are shown in Fig. S1.

2. Precipitation

Precipitation data were downloaded from: https://www.weather.gov/wrh/climate?wfo=sgx. 20 years of monthly mean precipitation (from 2000 to 2019) and 2020 precipitation in San Diego are shown in Fig. S2.

3. Nitrate data quality control

The nitrate data quality control process for data acquired from the Wirewalker (WW) SUNA V2 combination (Fig. S3 and text below) builds on previous quality control procedures for SUNA measurements (e.g., 1, 2):

- Step 1: The SUNA was configured to have a duty cycle of 20 minutes per hour with a sampling frequency of ca. 1 Hz. Raw nitrate data (unit: μM; 1 μM = 1 mmol m^{-3}) was recorded as a function of time. The 95% confidence interval of raw nitrate data (determined by calculating the probability density function of nitrate data within a 0.5 ^{o}C range) was 1 mmol m^{-3} (about 2 standard deviations). This value was used in subsequent error calculations.

- Step 2: The raw nitrate data contained outliers, such as unexpected zeros or extreme values (about 5% of the total amount of the data). These points were removed.

- Step 3: The SUNA V2 pump stream was oriented with the intake facing in an upwards direction. For this reason, only measurements collected during the upward, free-ascent travel of the Wirewalker were used in this analysis.

- Step 4: The nitrate data exhibited a temporal drift – increasing by ca. 3 mmol m^{-3} over 8 days consistently across all depths (Fig. S3a). This consistency indicates this temporal drift is an instrument error and thus needs to be accounted for. The minimum nitrate value within each duty cycle was identified. Then a low-pass filter with a cut-off frequency of 0.5 cpd was applied to the time series formed by these points to obtain a smoothed baseline. Assuming that the actual minimum nitrate concentration was consistently 0 over the course of the deployment, this baseline was subtracted from the upcast data to bring the minimum values back to 0, as shown in Fig. S3b.

- Step 5: The nitrate-temperature relationship using the temporal-drift-corrected nitrate data showed a positive relationship between nitrate and temperature above 12 ^{o}C (Fig. S3d, also appearing as an unrealistic non-monotonic vertical nitrate gradient near the ocean surface in Fig. S3b). Previous literature (e.g., 3) has also shown a temperature-dependent response of the nitrate sensor. This temperature effect was corrected by first fitting a linear function to the temperature and nitrate data in the temperature range where nitrate is expected to be 0 (here above 12 ^{o}C), and then subtracting this linear function from the data.

The result is shown in Fig. S3c-d, where nitrate is consistently 0 above a particular temperature threshold, as expected from previous data (e.g., 4).

- Step 6: Given the ca. 1 Hz sampling rate of SUNA, and an average vertical speed of 0.3 m s^{-1} for WW, nitrate data for each WW profile were gridded into uniform 1-m depth bins from the surface down to 100 m depth.

4. Chlorophyll fluorescence (ChlF) data analysis

Non-photochemical quenching (NPQ) is a photoprotective mechanism that suppresses fluorescence under high irradiance. Hence, *in situ* fluorescence data need to be corrected as NPQ-induced fluctuations do not represent real changes in biomass. Adapted from other NPQ correction methods applied to a diversity of profiling platforms, including the Wirewalker (e.g., 5-7), we estimated NPQ by first calculating the correlation coefficient between ChlF and PAR using all the data between the surface and 4 m in a two-day window. If the correlation coefficient was less than -0.1, the NPQ value for the full water column would be calculated as a linear function of PAR with the transfer function coefficients estimated from the linear fit of ChlF and PAR in the upper 4 m. The same process was applied incrementally to the whole data set using a 0.1-day offset. The final NPQ value was the average across all the increments that contained that time point. The NPQ-corrected ChlF data was obtained by adding the NPQ correction to the raw ChlF data.

ChlF was initially recorded in units of relative fluorescence units (RFU). To convert ChlF into the more common chlorophyll-a (Chl-a) concentrations (units: mg m^{-3}), a linear fit was calculated between the WW 31-33 m depth-averaged ChlF time series (RFU) and the nearby Del Mar Mooring 32 m calibrated Chl-a time series (mg m^{-3}) (correlation coefficient of 0.91, plot not shown here). This regression was used to estimate a conversion function for Chl-a from RFU to mg m^{-3}:

$$\boldsymbol{Chla} = \boldsymbol{0.05} * (\boldsymbol{ChlF} - \boldsymbol{44.95}) \qquad [1]$$

where $\boldsymbol{Chla}$ represents Chl-a in units of mg m^{-3} and $\boldsymbol{ChlF}$ represents ChlF in RFU. Eq.1 was applied to all the NPQ-corrected ChlF measurements.

5. The light field, with optical backscatter

The photosynthetically available radiation (PAR) was estimated by integrating the observed irradiance at wavelengths of 380 nm, 412 nm, 490 nm, and 532 nm and then interpolating/extrapolating to the range between 400 nm to 700 nm (i.e., the full PAR range). The equation is:

$$\boldsymbol{PAR(z,t)} = \sum_{n=1}^{4} \boldsymbol{I_n(z,t)\Delta\lambda} \times \frac{\mathbf{700-400}}{\mathbf{532-380}} \times \mathbf{0.046} \qquad [2]$$

where $\boldsymbol{I}$ is the measured irradiance in units of $\boldsymbol{\mu W\ cm^{-2}\ nm^{-1}}$, $\boldsymbol{n}$ represents each wavelength, $\boldsymbol{\Delta\lambda}$ is the wavelength interval size, and 0.046 is for conversion from $\boldsymbol{\mu W\ cm^{-2}}$ to $\boldsymbol{\mu mol\ m^{-2}\ s^{-1}}$. Fig. S4a shows that even though the surface PAR had some spikes, the shortwave radiation (measured independently at the Scripps Pier) was about two to ten times larger than surface PAR –- suggesting that about 50%-90% of the input solar energy was absorbed in the surface 3 m. Below the ocean surface, PAR was modeled as decaying exponentially in depth:

$$\boldsymbol{I(z)} = \boldsymbol{I_0 e^{-kz}}, \qquad [3]$$

where $\boldsymbol{I_0}$ is the PAR value at the ocean surface, $\boldsymbol{k}$ is the diffuse attenuation coefficient of irradiance, and $\boldsymbol{z}$ is depth. For each WW profile, $\boldsymbol{k}$ can be estimated from linear fits of $\boldsymbol{ln[I(z)]}$ vs. depth, giving

values between 0.08 m^{-1} and 0.6 m^{-1}. The euphotic depth (Z_{eu}), defined as the depth where PAR is 1% of its surface value, was estimated from:

$$Z_{eu} = \frac{-ln(0.01)}{k}. \quad [4]$$

A larger absolute Z_{eu} means that the water is clearer, with deeper penetration of light. A negative correlation between Z_{eu} and upper ocean (0-30 m) phytoplankton biomass (or particle load) (Fig. S4b) demonstrates that the subsurface light field was strongly modulated by upper ocean biological properties during the red tide: that is, the irradiance was attenuated (and the euphotic zone shoaled) by the dense phytoplankton community in addition to the expected decay of light in clear water.

Turbidity (suspended particle load) was estimated from the optical backscatter data using the instrument's factory calibration:

$$Turbidity = 0.0061 * (OBS - 50), \quad [5]$$

where $Turbidity$ is in NTU (Nephelometric Turbidity Units), and OBS is the measured optical backscatter at 532 nm. Fig. S4d showed similar spatial-temporal patterns as Chl-a (Fig. 1e), indicating that the suspended particles in the upper ocean were dominated by phytoplankton during the observational period. Note that we use turbidity as a relative measure, and the sensor is assumed to be stable in time.

6. Depth-to-isopycnal coordinate transformation

Raw data were recorded as a function of depth (pressure) and time, as seen in Fig. 1c-f. Within a depth range, raw measurements have variations both along and cross the isopycnals as a function of time. An example of nitrate concentrations in T/S space (Fig. S5) shows both a general trend of decreasing nitrate with decreasing density (i.e., cross-isopycnal variability), and variations of nitrate over time at the same density value (i.e., along-isopycnal variability). When internal waves were present, cross-isopycnal variability appears as temporal changes of nitrate (and other variables like temperature and salinity) in the depth coordinate system due to the vertical heaving of isopycnals. This complicates calculations of temporal changes at any depth, especially for dynamically passive but biologically active tracers such as nitrate. Transforming from depth coordinates to isopycnal coordinates removes the vertical displacements created by internal waves, decoupling the fluctuations induced by vertical displacements of isopycnals from those caused by biological uptake and horizontal (along-isopycnal) gradients.

The depth-to-isopycnal coordinate transformation was executed by interpolating raw physical and biogeochemical variables from each individual WW profile onto uniformly gridded density bins (here, the density interval was set to be 0.02 kg m^{-3}). Similarly, raw pressure measurements were also interpolated to give the instantaneous depths of each density bin for each profile. The mean isopycnal depth was calculated as the time-averaged interpolated depth for each gridded density bin over the course of the study period. As the mean isopycnal depth accounts for the different vertical spacings between different density layers, it provides a more realistic representation of the vertical distribution of density layers than using density as the y axis.

This different spacing of density bins in depth also leads to different numbers of raw measurement points in each gridded density bin, which results in different errors associated with the transformed values. To estimate the errors, the standard error of the mean (SEM; the standard deviation of the mean of multiple samples means) was calculated for each density bin:

$$SEM = \frac{\sigma}{\sqrt{n}}, \quad [6]$$

where $\boldsymbol{\sigma}$ is the standard deviation of the raw measurements, and $\boldsymbol{n}$ is the number of points within each density bin. An example of the coordinate transformation result is shown in Fig. S6, where vertical displacements of the isopycnals disappear after applying the depth-to-isopycnal transformation. While temperature and salinity are relatively constant along isopycnals (Fig. S6b,d), the along-isopycnal nitrate displays considerable variability (Fig. S6f).

7. Along-isopycnal velocities

As Eulerian measurements (fixed point mooring), the along-isopycnal WW time series for each variable contains temporal gradients driven by horizontal advection of existing spatial gradients. As seen in Fig. S7c, from noon 05/06 to 05/08, the along-isopycnal nitrate and horizontal velocities (southeast-ward current corresponding to decreasing of nitrate) were correlated, suggesting that changes in the along-isopycnal nitrate were partially driven by horizontal advection. Crucially, the advective signal will confound estimates of biologically driven nitrate rates of change, and thus must be accounted for. The detailed procedures are shown below.

8. Temperature/Salinity (T/S) binning

To separate the effects of horizontal advection from biological activities in the along-isopycnal nitrate time series, we applied a Temperature/Salinity binning method, which utilized spiciness as a tracer to follow the same water parcel (defined by the conservative tracers temperature and salinity) in time. Spiciness is a quantity related to Temperature/Salinity co-variability on a density layer: more positive means warmer and saltier water (spicier), while less positive means cooler and fresher water (mintier) (8). Thus, temporal changes in nitrate concentration driven only by biological processes can be estimated by calculating changes in nitrate concentration within each water mass. An example of how this method was performed is shown in Fig. S8. Along each isopycnal, data points were rearranged into evenly spaced spiciness bins (interval of 0.005 kg m^{-3}). Points in the same spiciness bin were assumed to represent the same water mass. Note that the number of data points in each spiciness bin will vary.

9. Estimation of Nitrate rate of change

Rates of change of nitrate, $\boldsymbol{\rho_{NO3}}$ (units: mmol N m^{-3} day^{-1}), quantify how fast nitrate changes over time, and are estimated from the specific nitrate rate of change, $\boldsymbol{r}$ (unit: day^{-1}), following the procedures described below.

After the T/S binning, within each water parcel, nitrate can be modeled as changing exponentially over time (similar to (9)). The equation for the temporal evolution of nitrate can be expressed as:

$$\boldsymbol{N(t) = N_0 e^{rt}}, \qquad [7]$$

where $\boldsymbol{N_0}$ is the initial nitrate concentration, $\boldsymbol{r}$ is the nitrate-specific rate of change of nitrate ($days^{-1}$), and $\boldsymbol{t}$ is time (days). Using consecutive time points in each spiciness bin (i.e., each water mass), $\boldsymbol{r}$ was estimated as:

$$\boldsymbol{r(i) = \frac{1}{t(i+1)-t(i)} * ln\left(\frac{N(i+1)}{N(i)}\right)}, \qquad [8]$$

where $\boldsymbol{N}$ is the measured nitrate concentration, and $\boldsymbol{i}$ represents the time index of the data. The error for $\boldsymbol{r}$ was calculated as:

$$\boldsymbol{r_E(i) = \frac{1}{t(i+1)-t(i)} * \sqrt{\left(\frac{SEM(i)}{N(i)}\right)^2 + \left(\frac{SEM(i+1)}{N(i+1)}\right)^2}}. \qquad [9]$$

An example is shown in Fig. S9a. Note that this exponential fitting was only applied when the absolute difference between two points was larger than 2 mmol m^{-3} (2 times raw nitrate data's confidence limit) — ensuring a statistically significant estimate of r that can be further interpreted as biological processes. Due to this criterion, some estimates of r were undefined. Overall, the probability density functions (PDFs) of the estimated $\boldsymbol{r}$ (Fig. S9d) show a clear distribution of the majority of the data being negative, consistent with the observations of nitrate loss over time. Furthermore, estimates of $\boldsymbol{r}$ give a distinct mode value of -1 day^{-1} (Fig. S9d), indicating that the biologically driven loss rate of nitrate is relatively constant among different water parcels, across different isopycnals, and over time. Although experiments were done under different conditions (lab vs. nature), and with different species (*Heterocapsa niei* vs. *Lingulodinium polyedra*), our estimated $\boldsymbol{r}$ is consistent with the values reported in (9).

A temporal integration of the estimated $\boldsymbol{r(z,t)}$ was performed to estimate the changes in the nitrate field driven only by biological activities – that is, without the effects of advection. Due to the fact that estimates of r had uneven time intervals along each isopycnal (primarily due to the inconsistent presence of different water masses at the WW), a rebinning in time was executed by setting a uniform time bin of 4 hours, and choosing the median $\boldsymbol{r}$ value within each 4-hour window to be the rebinned $\boldsymbol{r}$, denoted as $\boldsymbol{r_{bin}}$. Its error is:

$$\boldsymbol{r_{bin,E}} = \frac{\boldsymbol{1}}{\boldsymbol{m}} * \sqrt{\sum_{\boldsymbol{i=1}}^{\boldsymbol{m}} (\boldsymbol{r_E(i)})^2}, \qquad [10]$$

where $\boldsymbol{m}$ is the number of estimates of $\boldsymbol{r}$ in each time bin. Even though the time resolution of the nitrate estimates decreased after this rebinning process, there were two distinct advantages: 1) the process generated a uniform time interval for further nitrate calculations; 2) the process compensated for non-significant values previously excluded in the estimates of $\boldsymbol{r}$.

The predicted biologically mediated nitrate concentration, $\boldsymbol{N_{pred}}$ was calculated using the estimates of $\boldsymbol{r}$ along each isopycnal as:

$$\boldsymbol{N_{pred}(i+1)} = \boldsymbol{N_{pred}(i)} e^{\boldsymbol{r_{bin}(i) * \Delta t}}, \qquad [11]$$

where $\boldsymbol{\Delta t}$ is the time interval between estimates of $\boldsymbol{r}$ (4 hours), $\boldsymbol{i}$ represents the index of the data, and the initial value is taken from the first profile shown in Fig. 2c from the observations. The error associated with the predicted nitrate is estimated as:

$$\boldsymbol{N_{pred,E}(i+1)} = \sqrt{(e^{\boldsymbol{r_{bin}(i)*\Delta t}} * \boldsymbol{N_{pred,E}(i)})^2 + (\boldsymbol{N_{pred}(i)} * \boldsymbol{\Delta t} * e^{\boldsymbol{r_{bin}(i)*\Delta t}} * \boldsymbol{r_{bin,E}(i)})^2}\,. \qquad [12]$$

$\boldsymbol{N_{pred}}$ is shown in Fig. S9b, with signatures of clear deepening of nitrate contours in time, and also decreasing of nitrate along isopycnals in time between 20 m and 33 m. These patterns are consistent with the observations (Fig. 2c), but have had advection-associated fluctuations removed.

To estimate the rate of change of nitrate, $\boldsymbol{\rho_{NO3}}$, a linear differentiation in time was applied to the predicted nitrate concentrations (Fig. S9b) along each isopycnal:

$$\boldsymbol{\rho_{NO3}(i)} = \frac{\boldsymbol{d\,N_{pred}(i)}}{\boldsymbol{dt}} = \frac{\boldsymbol{N_{pred}(i)} * [e^{\boldsymbol{r_{bin}(i)*\Delta t}} - \boldsymbol{1}]}{\boldsymbol{\Delta t}}. \qquad [13]$$

The associated error was estimated by:

$$\boldsymbol{\rho_{NO3,E}} = \sqrt{(\frac{[e^{\boldsymbol{r_{bin}*\Delta t}} - \boldsymbol{1}]}{\boldsymbol{\Delta t}} * \boldsymbol{N_{pred,E}})^2 + (\boldsymbol{N_{pred}} * e^{\boldsymbol{r_{bin}*\Delta t}} * \boldsymbol{r_{bin,E}})^2}. \qquad [14]$$

A modal value of -6 mmol m^{-3} day^{-1} was obtained from the PDF of $\boldsymbol{\rho_{NO3}}$ (Fig. S9e), which is consistent with previous literature (1 - 4 mmol m^{-3} day^{-1}, with *L. polyedra* concentrations of 10^5 cells

per liter, 10, 11). The estimated nitrate loss ($\boldsymbol{N_{loss}}$) was simply obtained by integrating $\boldsymbol{\rho_{NO3}}$ in time with a zero initial value, and had the same error bars as $\boldsymbol{N_{pred,E}}$. Generally, the error bars for depth integrals, $\boldsymbol{E_D}$, can be expressed as:

$$\boldsymbol{E_D} = \sqrt{\sum_{i=1}^{Q}(\boldsymbol{dz}(\boldsymbol{i}) * \boldsymbol{err}(\boldsymbol{i}))^2}\ , \qquad [15]$$

where $\boldsymbol{Q}$ is the number of density layers, $\boldsymbol{dz}$ is the depth difference between each layer, and $\boldsymbol{err}$ is the error bar of the integrated variable.

In all, these approaches allow us to accurately decouple vertical and horizontal advection signals from biologically driven changes of the nitrate field — even though we had only a single mooring and so could not directly estimate horizontal spatial gradients. The results produce features in the data that are consistent with other independent data (PAR, Chl-a, turbidity, etc.), further supporting the feasibility of this T/S binning method; this method could potentially be applied to other research problems in similar situations.

Finally, to emphasize how important it is to remove horizontal advection when estimating nitrate uptake rate, we compare the depth-integrated nitrate loss calculated from the raw observational nitrate data to the “de-advected” nitrate data ($\boldsymbol{N_{pred}}$) (Fig. S10). This comparison shows clearly that there are large excursions in nitrate concentration in the raw nitrate data (the blue curve), which are not seen in the “de-advected” data (red line). Our calculations show that these fluctuations were driven by horizontal advection of horizontal, along-isopycnal gradients of nitrate.

Supporting figures

Fig. S1

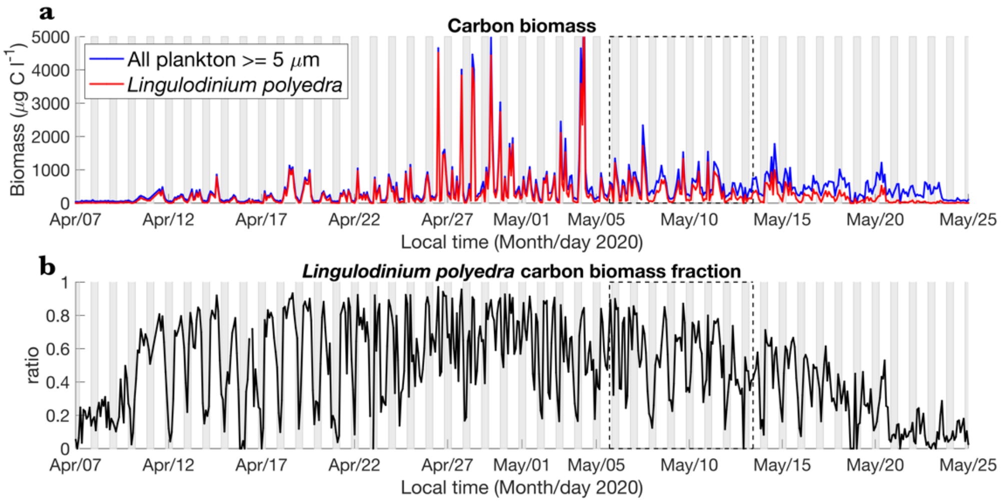

Figure S1. IFCB-estimated carbon biomass over the entire bloom. a) carbon biomass of *L. polyedra* (red) and all plankton that were ≥5 µm but within IFCB's upper size limit (blue). b) carbon biomass of *L. polyedra* as a fraction of the carbon biomass of all imaged plankton ≥5 µm. The grey vertical bars indicate nighttime. The dashed black box represents our study period.

Fig. S2

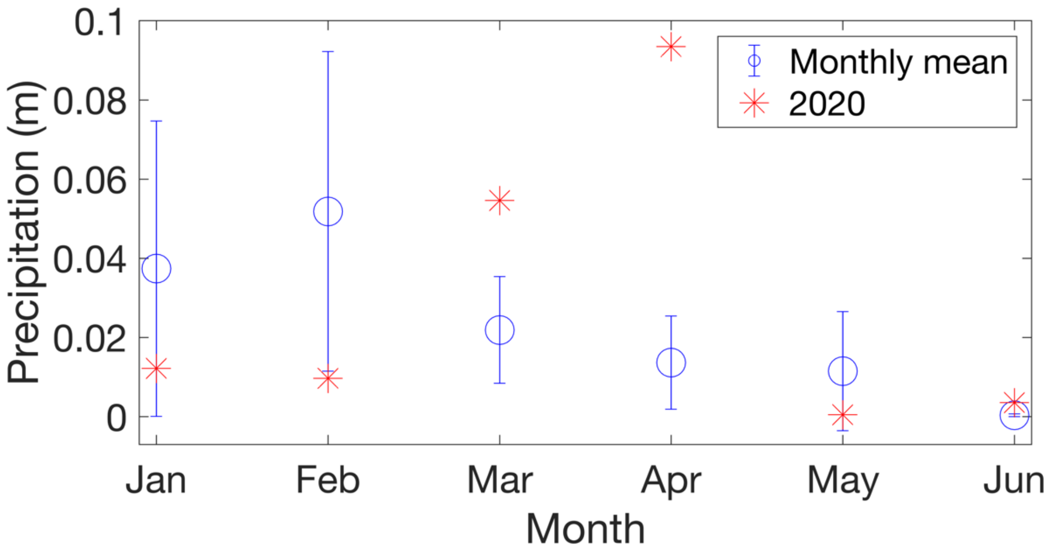

Figure S2. Monthly mean precipitation (from 2000 to 2019) vs. 2020 precipitation in San Diego. The error bars are one standard deviation. Data from the National Weather Service.

Fig. S3

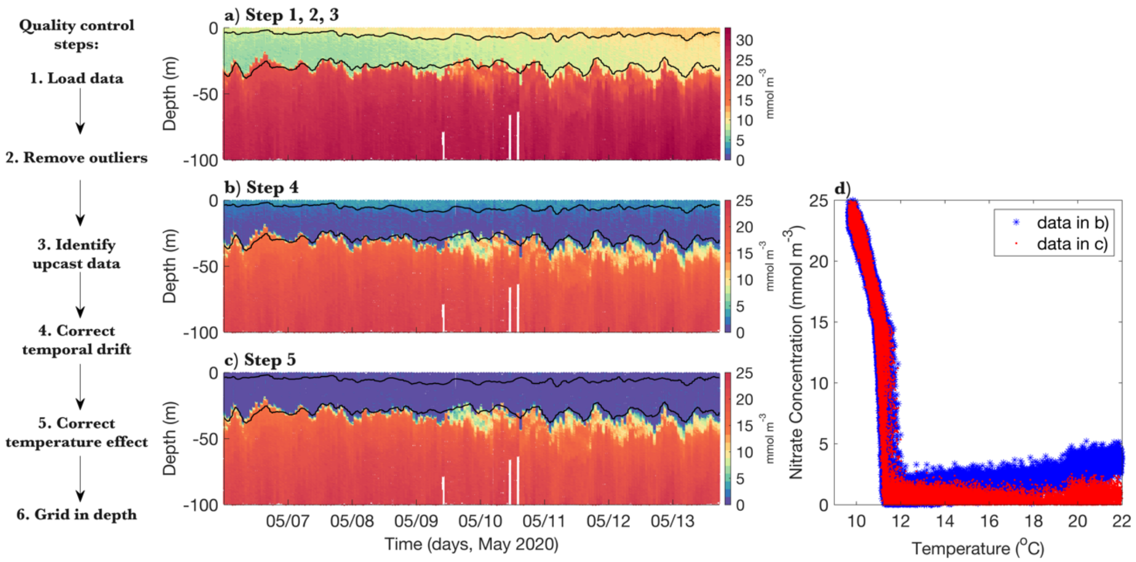

Figure S3. Nitrate data quality control procedures. Text on the left outlines the steps for nitrate data quality control. a-c) The results after specific step(s) have been applied to the nitrate data, with nitrate plotted in color as a function of depth and time. The two black lines are two isopycnals plotted for reference. Note that the colorbar range is different in a, compared to b and c, but the colormap is the same from 0-25 mmol m^{-3} for all panels. d) The nitrate-temperature relationship based on data before (blue stars) and after (red dots) implementing the temperature correction.

Fig. S4

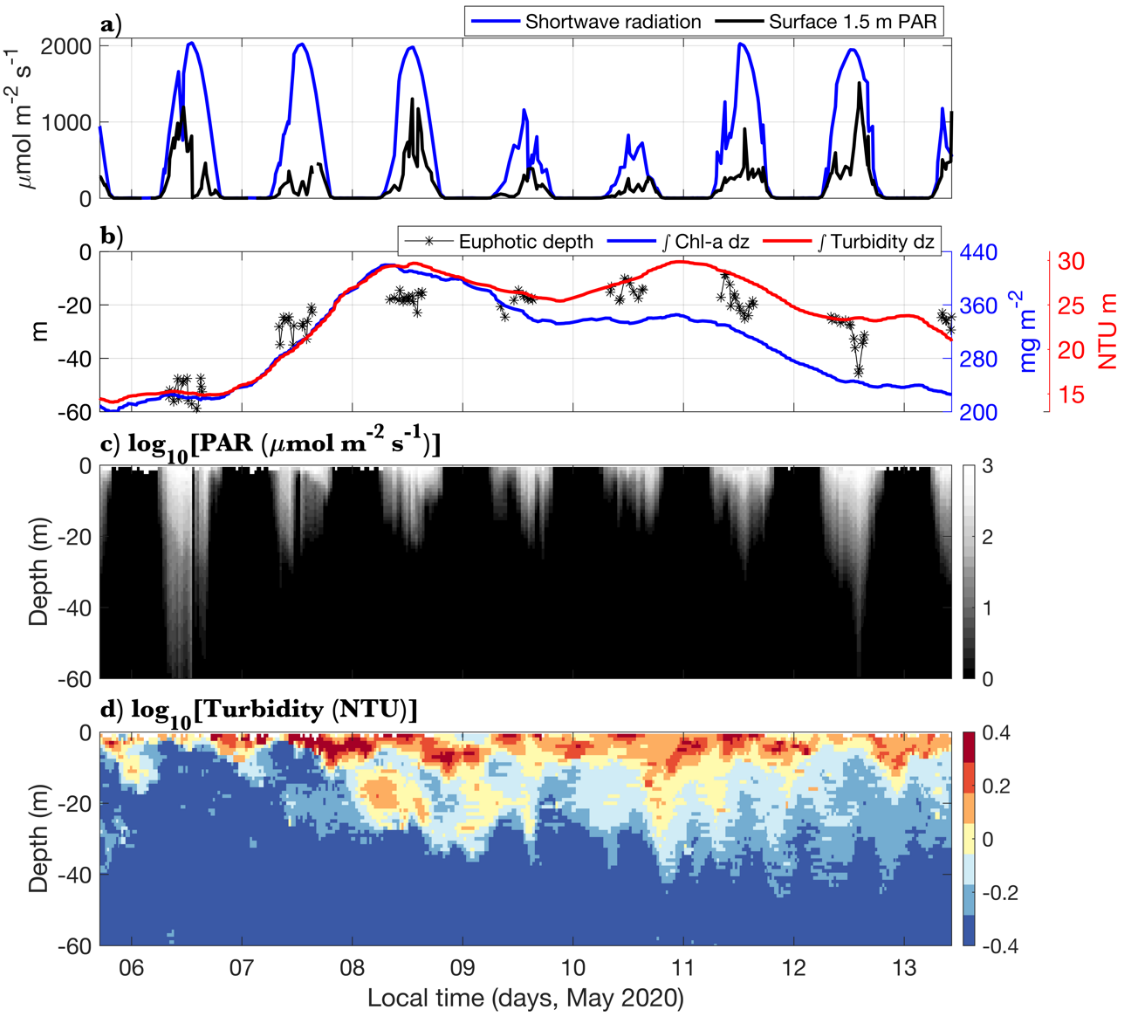

Figure S4. Irradiance and turbidity. a) Shortwave radiation (400 nm - 700 nm range) measured at the Scripps Pier (blue line) and surface 3 m depth-averaged PAR estimated from the irradiance measurements from the Wirewalker (black line). b) Estimated euphotic depth (black stars), and 0-30 m depth-integrated Chl-a and turbidity in blue and red respectively. Note that the Chl-a and turbidity time series have been filtered a 24-h running mean to smooth out short-time-scale variability. c-d) PAR and turbidity from the WW as a function of time and depth, respectively. Note that only the upper 60 m of data are shown here.

Fig. S5

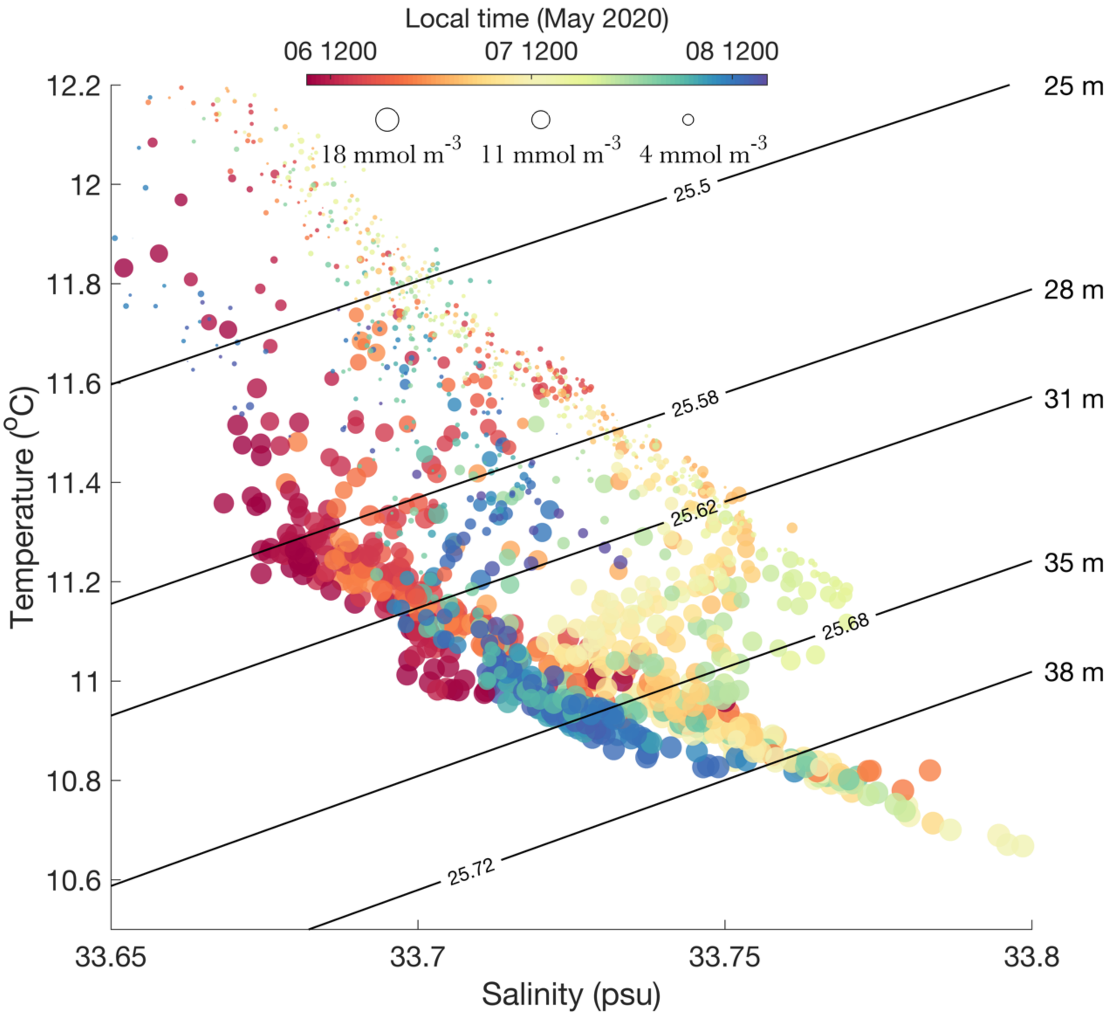

Figure S5. Temperature and salinity characteristics of data (T/S diagram) from the Wirewalker acquired during a 2-day window between 22 and 35 m depth. Data points are colored by time, and point size is proportional to nitrate concentration. The black lines are five isopycnals, with their corresponding mean isopycnal depths denoted at the right-hand end of the curve. Note that there is a variation of T, S, and nitrate along some isopycnals.

Fig. S6

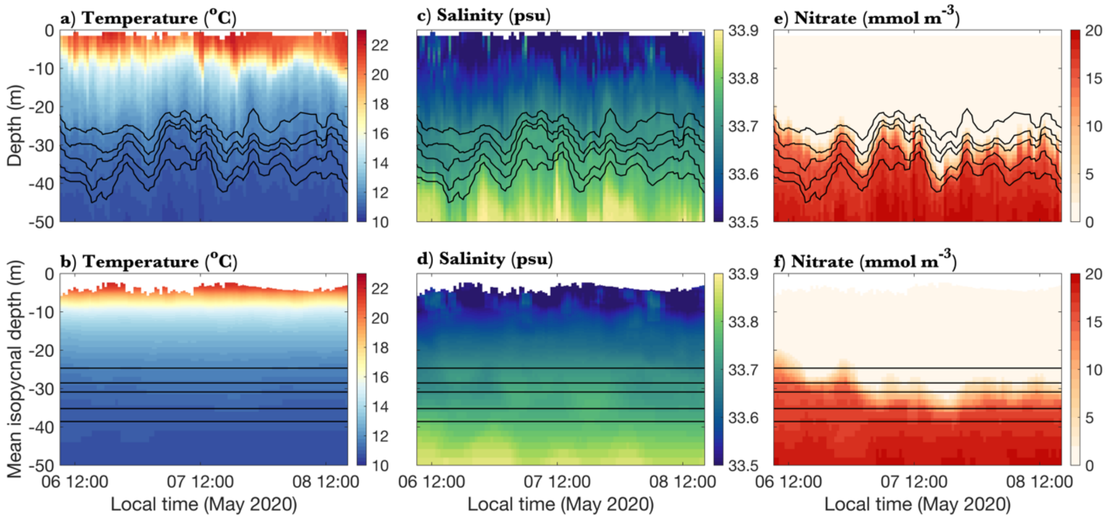

Figure S6. Examples of the depth-to-isopycnal coordinate transformation. a/c/e) Observed temperature, salinity, and nitrate as a function of depth and time. Black lines show isopycnals with values of 1025.5, 1025.58, 1025.62, 1025.68, and 1025.72 kg m^{-3} respectively. b/d/f) Temperature, salinity, and nitrate as a function of gridded density bin (plotted as their mean depths) and time, with black lines representing the same densities as the upper panels.

Fig. S7

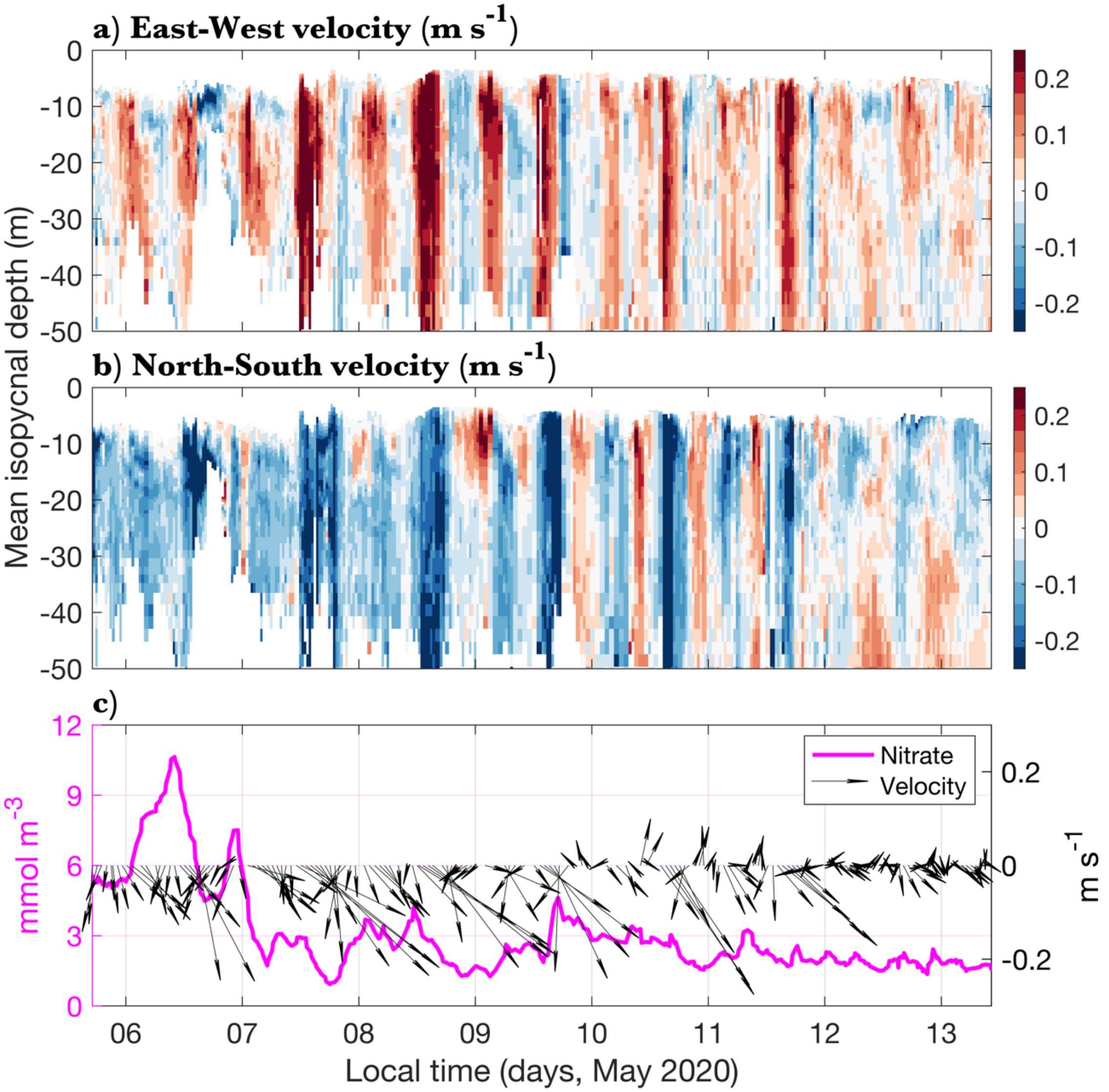

Figure S7. a-b) Along-isopycnal East-West velocity and North-South velocity respectively. c) 20-35 m depth-averaged nitrate in magenta, with black arrows showing the direction and the magnitude of the velocity, with up and right indicating the northward and eastward direction, respectively. Note the correlations of nitrate fluctuations with velocity fluctuations, consistent with horizontal advection of existing horizontal (along-isopycnal) gradients of nitrate.

Fig. S8

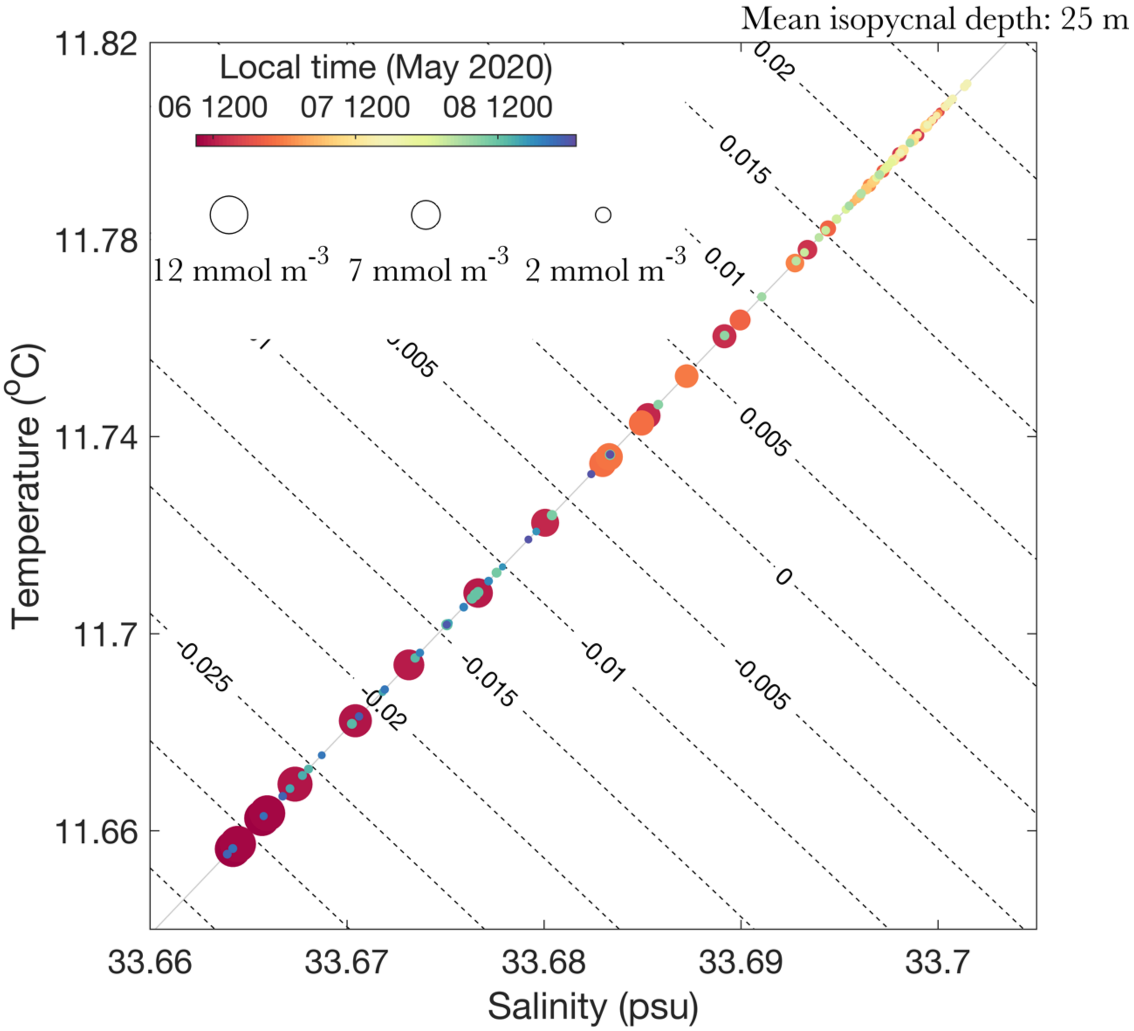

Figure S8. An example of T/S binning by spiciness on the 1025.5 kg m^{-3} isopycnal. Color represents time, and symbol size represents nitrate concentration. The grey line shows the isopycnal. The black dashed lines are spiciness anomaly contours (spiciness minus the spiciness calculated from the mean T/S on this isopycnal) with an interval of 0.005 kg m^{-3}.

Fig. S9

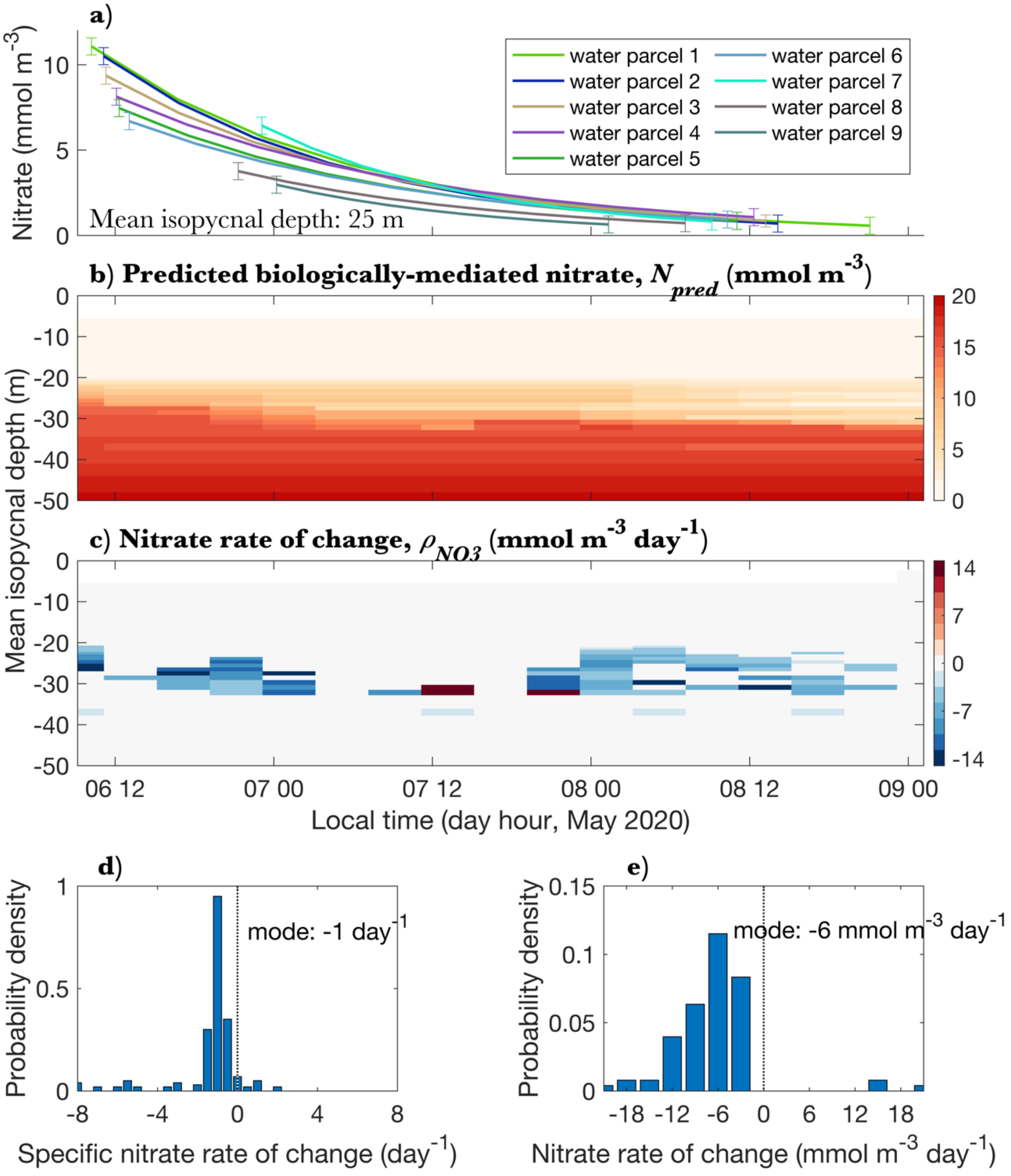

Figure S9. Estimation of the nitrate rates of change. a) To estimate $\boldsymbol{r}$, exponential curves were fit to the data points for each water parcel on an isopycnal determined by the T/S binning. b,c) The predicted nitrate field, and estimated nitrate rate of change $\boldsymbol{\rho_{NO3}}$, respectively. d,e) The probability density functions of $\boldsymbol{r}$, and $\boldsymbol{\rho_{NO3}}$, respectively.

Fig. S10

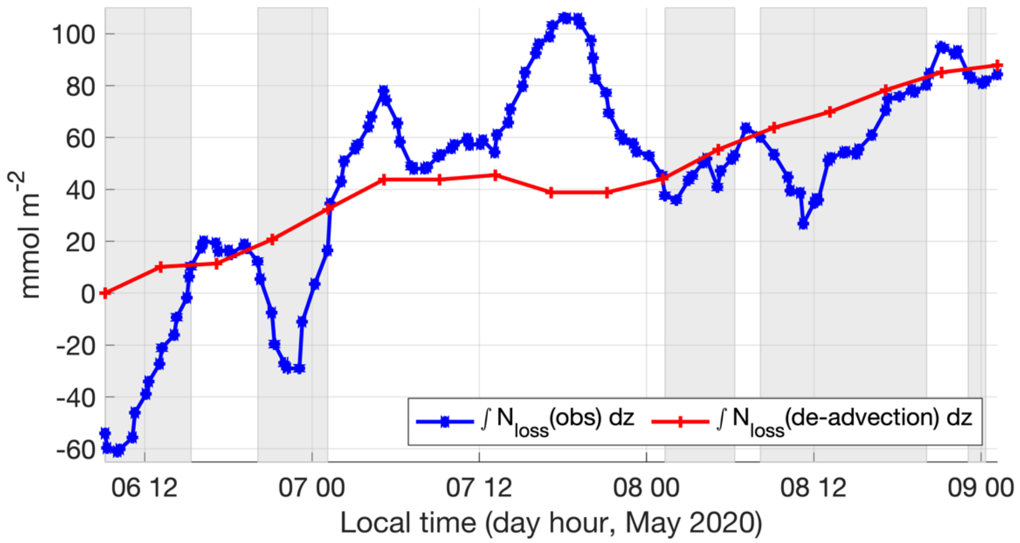

Figure S10. Comparison between depth-integrated nitrate loss based on the raw observed nitrate data (blue line), and the de-advected nitrate data (red line). Grey areas represent periods when the blue line is less than the red line, indicating times when nitrate decreased due to advection, not biological uptake.

SI References

1. L. E. Lilly *et al*., Biogeochemical anomalies at two southern California Current System moorings during the 2014–2016 Warm Anomaly-El Niño sequence. *Journal of Geophysical Research: Oceans*, *124*(10), pp.6886-6903 (2019).
2. K. S. Johnson *et al*., BGC-Argo quality control manual for nitrate concentration (2021).
3. C. M. Sakamoto, K. S. Johnson, L. J. Coletti, Improved algorithm for the computation of nitrate concentrations in seawater using an in situ ultraviolet spectrophotometer. *Limnology and Oceanography: Methods*, *7*(1), pp.132-143 (2009).
4. A. J. Lucas, *et al*., The green ribbon: Multiscale physical control of phytoplankton productivity and community structure over a narrow continental shelf. *Limnology and Oceanography*, *56*(2), pp.611-626 (2011).
5. R. E. Davis, M. D. Ohman, D. L. Rudnick, J. T. Sherman, Glider surveillance of physics and biology in the southern California Current System. *Limnology and Oceanography*, *53*(5part2), pp.2151-2168 (2008).
6. R. E. Todd, D. L. Rudnick, R. E. Davis, Monitoring the greater San Pedro Bay region using autonomous underwater gliders during fall of 2006. *Journal of Geophysical Research: Oceans*, *114*(C6) (2009).
7. T. Schlosser, A. J. Lucas, M. Omand, T. Farrar, Monsoons, plumes, and blooms: intraseasonal variability of subsurface primary productivity in the Bay of Bengal. [Manuscript submitted for publication, 2023].
8. P. Flament, A state variable for characterizing water masses and their diffusive stability: Spiciness. *Progress in Oceanography*, *54*(1-4), pp.493-501 (2002).
9. J. J. Cullen, Diel vertical migration by dinoflagellates: roles of carbohydrate metabolism and behavioral flexibility. *Contrib. Mar. Sci*, *27*, pp.135-152 (1985).
10. R. W. Eppley, W. G. Harrison, "Physiological ecology of Gonyaulax polyedra, a red water dinoflagellate off southern California" In, *Proc. 1st conf. toxic dinoflagellate blooms*, edited by V. LoCicero, M.S.T.F., Wakefield, Mass., pp. 11-22 (1975).
11. W. G. Harrison, Nitrate metabolism of the red tide dinoflagellate *Gonyaulax polyedra* Stein. *Journal of Experimental Marine Biology and Ecology*, *21*(3), pp.199-209 (1976).
